# Circulating Orexin-A Levels in Patients With Schizophrenia, Bipolar Disorder, and Major Depressive Disorder: A Systematic Review and Meta-Analysis

**DOI:** 10.7759/cureus.101525

**Published:** 2026-01-14

**Authors:** Shatha Almahwzi, Samer Ghabashi, Wadha Alyami, Jana Alsolami, Rashid Juma, Sarah Alghamdi, Lojean Althobyane, Manar Alqahtani, Meshal Alotaibi

**Affiliations:** 1 College of Medicine, Ibn Sina National College for Medical Studies, Jeddah, SAU; 2 College of Medicine, Trinity College Dublin, Dublin, IRL; 3 College of Medicine, Princess Nourah Bint Abdulrahman University, Riyadh, SAU; 4 College of Medicine, King Abdulaziz University Faculty of Medicine, Jeddah, SAU; 5 College of Medicine, Alfaisal University College of Medicine, Riyadh, SAU; 6 College of Medicine, Al Baha University, Al Baha, SAU; 7 College of Medicine, Taibah University, Madinah, SAU; 8 College of Medicine, King Saud Bin Abdulaziz University for Health Sciences College of Medicine, Riyadh, SAU; 9 Department of Adult Mental Health, King Abdulaziz Medical City Riyadh, Riyadh, SAU

**Keywords:** bipolar disorder, major depressive disorder, orexin-a, schizophrenia, systematic review

## Abstract

Orexin-A, a neuropeptide involved in regulating physiological processes, has potential implications in psychiatric disorders. Studies examining orexin-A levels in schizophrenia, bipolar disorder, and major depressive disorder have produced conflicting results, highlighting the need for further research. To address these inconsistencies, this meta-analysis aims to synthesize existing data on orexin-A levels across these disorders, clarifying its role and potential as a diagnostic or prognostic biomarker.

This systematic review, registered with PROSPERO, investigated plasma orexin-A levels in schizophrenia, bipolar disorder, and major depressive disorder. Relevant studies were identified through PubMed and PsycINFO using specific keywords. Inclusion criteria targeted adult patients with these disorders and healthy controls, assessing quality and bias using the MINORS tool for non-randomized studies and the AXIS tool for cross-sectional studies. A meta-analysis compared orexin-A levels between groups, using random-effects models and evaluating statistical heterogeneity and publication bias.

Of the 64 reviewed articles in full-text screening, 11 met the criteria for analysis, involving 1,778 participants (514 with schizophrenia, 149 with bipolar disorder, and 461 with major depressive disorder). The meta-analysis revealed significantly decreased plasma orexin-A levels in schizophrenia (SMD = -0.38; 95% CI: -0.72 to -0.05; p = 0.03) and increased levels in major depressive disorder (SMD = 0.38; 95% CI: 0.15 to 0.60; p = 0.001) compared to healthy controls. Moderate statistical heterogeneity was noted in the schizophrenia analysis (I^2 = 54%). Methodological quality was generally high; MINORS scores ranged from 12 to 20 (out of 24), and AXIS scores ranged from 17 to 19 (out of 20), indicating a moderate-to-low risk of bias.

These findings suggest distinct orexin-A dysregulation in schizophrenia and major depressive disorder. However, findings in bipolar disorder were inconclusive due to high heterogeneity, indicating orexin-A’s complex involvement in mood and energy regulation.

## Introduction and background

Orexin, a neuropeptide that circulates in cerebrospinal fluid (CSF), plays a role in wakefulness, arousal, regulation of circadian rhythm, appetite, and cognitive function [1]. In the lateral hypothalamus, orexin is produced in two main forms: orexin-A and orexin-B (hypocretin-1 and hypocretin-2), which bind to orexin-1 and orexin-2 receptors, respectively, with considerable affinities [2].

Measuring orexin-A in cerebrospinal fluid (CSF) is essential for the diagnosis of narcolepsy type one, which is characterized by very low levels of orexin-A, as a value below 110 pg/ml is required to establish the diagnosis [3]. Radioimmunoassay (RIA) is a standard method with high specificity and sensitivity in orexin quantification [4]. Additional methods for orexin measurements include enzyme immunoassay (EIA) and fluorescence immunoassay (FIA) [5,6].

Orexin dysregulation was observed among psychiatric patients, highlighting orexin’s potential involvement in the pathophysiology of psychiatric disorders [1]. Several studies have investigated the orexins' role in schizophrenia and mood disorders [7-11]. For example, animal models of depression observed a reduction in the number and size of orexin neurons [7]. Additionally, depressive behavior was significantly reduced following intracerebral injection of orexin-A [8]. In human studies, polymorphism in the HCRTR1 gene, a gene that encodes orexin-1 receptors, was positively associated with major depressive disorder [9]. Brundin L et al. reported that patients with major depressive disorder had lower orexin-A levels [10]. However, another study revealed that orexin-A levels were higher in patients with depression compared to healthy controls [11]. 

In bipolar disorder (BP), evidence indicates that patients exhibit reduced orexin-A levels relative to controls [12]. Conversely, another study reported elevated orexin-A levels in patients with bipolar disorder compared to controls [13].

Altered orexin-A level was observed among schizophrenia patients and was linked to symptoms and treatment response. For example, a significant positive correlation has been found between orexin levels and sleep latencies in schizophrenia patients [14]. Furthermore, orexin-A level could be used to predict response to clozapine. Clozapine is an atypical antipsychotic medication that is used to treat resistant schizophrenia [15,16]. Elevated orexin-A level was significantly associated with clozapine response, as a study documented that the clozapine-responsive group has a high orexin-A level compared to the clozapine-resistant and antipsychotic-free groups [17]. Another study by Tsuchimine et al. reported that a subgroup of people with schizophrenia has higher levels of orexin-A than normal controls [12]. Furthermore, patients with schizophrenia with higher orexin-A levels had fewer negative and disorganized symptoms [18]. Conversely, a systematic review and meta-analysis deny observation of any abnormality in orexin-A concentration in patients with schizophrenia [19]. As these results seem contradictory, a comprehensive study is needed to overcome these inconsistencies. This study aims to investigate the relationship between plasma orexin-A levels in adult patients with schizophrenia, bipolar disorder, and major depressive disorder compared to healthy controls.

## Review

Methodology

Review of the Literature

This systematic review was conducted following the Preferred Reporting Items for Systematic Reviews and Meta-Analyses (PRISMA) guidelines [20]. The study protocol was registered with PROSPERO with the following ID: CRD42024570341 [21]. Due to the nature of the study, ethical approval was not required. Relevant studies were identified in July 2024 by using the electronic databases of PubMed, PsycINFO, Web of Science, Embase, Google Scholar, and Cochrane Library. The following keywords were used: (Schizophrenia OR schizophrenic disorders OR dementia praecox OR bipolar disorders OR bipolar mood disorder OR depression OR depressive disorder) AND (orexin OR hypocretin OR orexin-A OR hypocretin-1 OR orexin-B OR hypocretin-2) AND (plasma OR serum) AND (measurement OR levels). The search was compliant with the PICO criteria [22]. To ensure the inclusion of the most recent evidence, an updated search was conducted in July 2025 using the same search strategy, focusing on PubMed and Google Scholar to identify any newly published studies. However, no additional studies met the inclusion criteria and were therefore not included in the final analysis. Deviations from the registered PROSPERO protocol included (1) the exclusion of bipolar disorder and certain subgroup analyses (antipsychotic medications in schizophrenia) from the quantitative meta-analysis due to extreme heterogeneity (I^2 > 90%) observed during preliminary analysis and (2) the decision to report results using standardized mean difference (SMD) rather than mean difference (MD) to account for variability in assay calibration and unit reporting (e.g., pg/mL vs. ng/L) across the included studies.

Inclusion and Exclusion Criteria

This systematic review included (a) adult patients diagnosed with schizophrenia, MDD, or bipolar disorder (DSM-IV/V or ICD-10); (b) studies with healthy control groups; (c) studies measuring plasma orexin-A levels; (d) randomized controlled trials or observational studies (cross-sectional, cohort, case-control); and (e) studies published in English.

Studies were excluded if they (a) involved participants with comorbid physical or neurological conditions known to independently alter orexin levels (e.g., narcolepsy, traumatic brain injury, or severe metabolic disorders) or (b) were non-original research articles, including reviews, meta-analyses, editorials, case reports, or conference abstracts.

Assigned reviewers independently screened each eligible study, and disagreements were resolved through discussion after retrieving the full text to determine whether inclusion and exclusion criteria were met.

Screening and Data Extraction

Three independent reviewers (SA, JA, and SMA) screened papers simultaneously and independently and reviewed them by title and abstract using the Rayyan search web and mobile app for systematic review [23]. The full text was then reviewed by three independent reviewers (MA, RJ, and WA) simultaneously. Data extraction was performed by two reviewers (SA and LA) for the following variables:

Study characteristics: first author name, publication year, country where the study was performed, and the study design, age, gender, body mass index, smoking, and medication status. 

Primary outcome: the mean, standard deviation, and sample size of plasma orexin-A levels for patient and control groups. 

Secondary outcomes: Positive and Negative Syndrome Scale (PANSS), Hamilton Depression Rating Scale (HAM-D), Brief Psychiatry Rating Scale (BPRS), Young Mania Rating Scale (YMRS), and Pittsburgh Sleep Quality Index (PSQI). To avoid duplication, the retrieved data were double-checked independently by two separate reviewers.

Assessment of Quality and Bias Risk

The methodological quality of the included studies was assessed using tools specific to their study designs.

For cross-sectional studies, the Appraisal tool for Cross-Sectional Studies (AXIS) was employed. This 20-item tool evaluates study design, target population, sampling, and reporting quality. Each item is scored as 1 (Yes) or 0 (No/Don’t Know), yielding a total score that indicates the overall quality of reporting and risk of bias.

For non-randomized studies (case-control and cohort): The Methodological Index for Non-Randomized Studies (MINORS) was used [24]. This instrument consists of 12 items for comparative studies, with each item scored from 0 to 2 (0 = not reported, 1 = reported inadequately, 2 = reported adequately). The total score ranges from 0 to 24 for comparative studies, with higher scores reflecting higher methodological quality. Two reviewers (RJ and WA) independently assessed each study, and disagreements were resolved through discussion.

Meta-Analysis of the Included Data

Statistical analyses were performed using Review Manager (RevMan) [Computer program], Version 5.4, The Cochrane Collaboration (Copenhagen, Denmark). The standardized mean difference, or weighted mean difference, with 95% confidence intervals, was calculated to compare the plasma orexin-A levels between patient and control groups. A random-effects model was employed to account for potential heterogeneity between studies. Also, potential differences between mental health disorders were examined by subgroup analyses by diagnostic category (schizophrenia, major depressive disorder, and bipolar disorder). Moreover, the statistical heterogeneity was evaluated using Cochrane's Q test and the I-squared (I²) statistic. To explore sources of heterogeneity and conduct subgroup analyses and meta-regression, considering study design, patient characteristics, and methodological factors. Assessment of publication bias using funnel plots was planned. However, due to the limited number of studies included in each subgroup analysis (n < 10), these tests were not performed, as they would lack sufficient statistical power to detect asymmetry. Sensitivity analyses were performed to assess the robustness of the findings, such as excluding studies with a high risk of bias or influential studies.

Results 

*The Literature Findings* 

**Figure 1:**
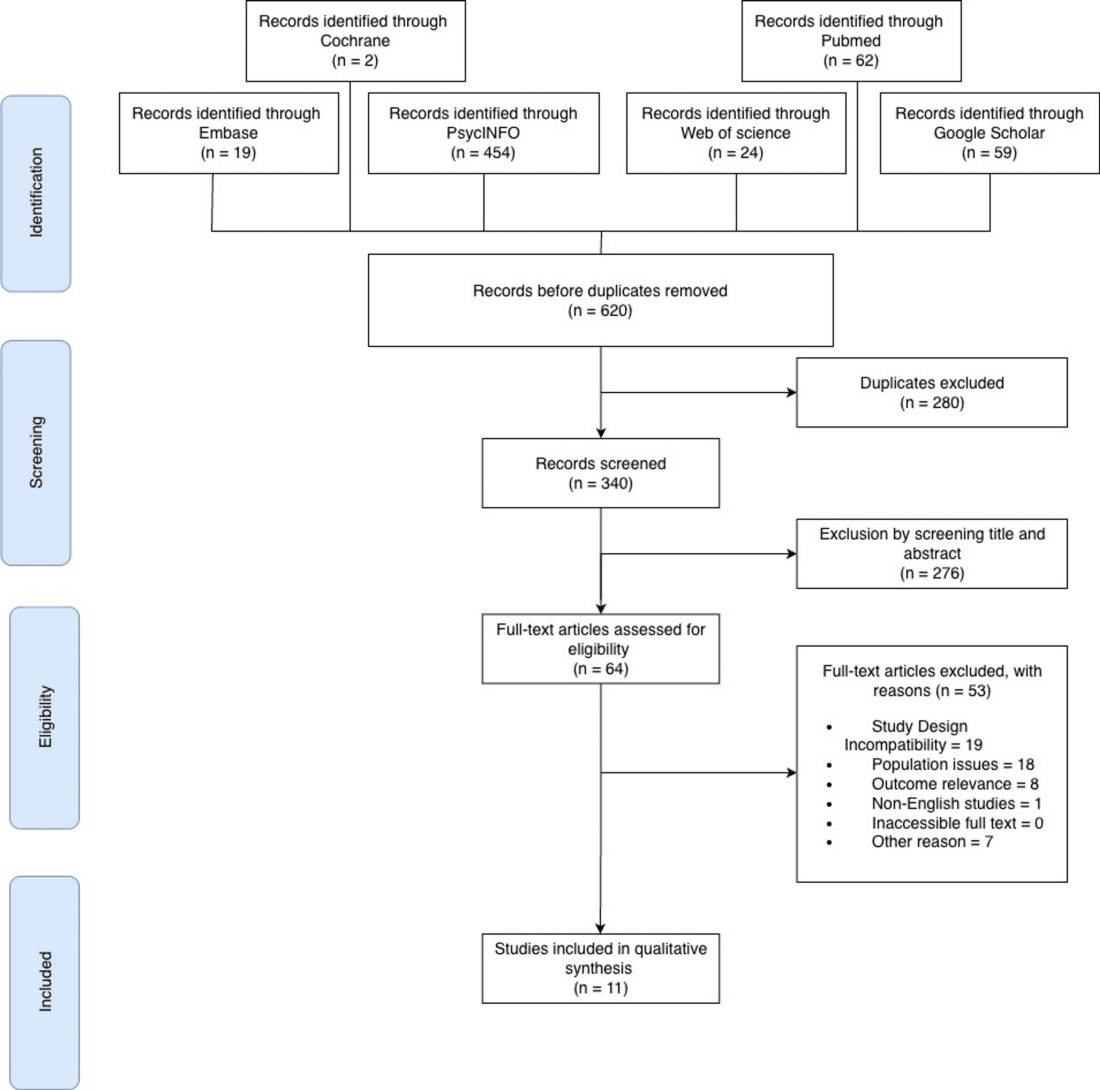
PRISMA diagram

In this study, a total of 620 articles were initially collected. After removing 280 duplicates, the remaining studies were screened for eligibility. These articles were sourced from several databases, including PubMed, Google Scholar, Embase, PsycINFO, Web of Science, and the Central Register of Controlled Clinical Trials (CENTRAL). Initially, the full texts of 64 articles were reviewed. However, after applying the exclusion criteria, only 11 articles published between January 2010 and March 2024 were included in the final analysis in Figure 1 [11-13,18,25-31]. Among these, there were two case-control studies, two prospective cohort studies, and seven cross-sectional studies. The characteristics of all the articles included are presented in Table 1.

**Table 1:** A description of the characteristics of the included studies Notes: No., Number of Participants; BMI, Body Mass Index; a BMI for Depression with Childhood Trauma (CT) group; b BMI for Depression without Childhood Trauma (CT) group; M:F refers to the gender distribution; BMI is measured by kg/m². A dash (-) indicates that data were unavailable or not reported.

	Healthy group				Schizophrenia group				Bipolar group				Depression group			
Study ID	No.	Age/Mean (±SD)	BMI	M:F	No.	Age/Mean (±SD)	BMI	M:F	No.	Age/Mean (±SD)	BMI	M:F	No.	Age/Mean (±SD)	BMI	M:F
Basoglu, 2010 [29]	22	21.7 ± 1.1	22.4 ± 2.0	-	22	21.2 ± 0.75	-	-	-	-	-	-	-	-	-	-
Sun, 2016 [28]	15	22.20 ± 2.14	-	-	13	18-45	-	-	-	-	-	-	-	-	-	-
Lu, 2021 [25]	82	27.33 ± 9.41	-	1:1.16	13	-	-	27:34:00	-	-	-	-	-	-	-	-
Wang, 2023 [31]	51	29.35 ± 8.41	22.6 kg/m²	26(M):25(F)	-	-	-	-	-	-	-	-	97	33.56 ± 14.11	22.18ᵃ / 24.22ᵇ	33(M); 64(F)
Li, 2021 [13]	82	27.32 ± 9.40	-	38(M):44(F)	-	-	-	-	57	25.04 ± 6.56	-	30(M); 27(F)	74	27.58 ± 7.56	-	32(M); 42(F)
Tsuchimine, 2019 [12]	80	47.0 ± 14.2	21.9 kg/m²	32(M); 48(F)	80	36.8 ± 11.2	23.2 kg/m²	38(M); 42(F)	40	41.1 ± 12.1	23.4 kg/m²	16(M); 24(F)	80	43.7 ± 10.4	22.7 kg/m²	37(M); 43(F)
Yu, 2023 [26]	54	27.94 ± 9.55	20.95 kg/m²	23:31	54	22.02 ± 7.25	20.67 kg/m²	26:28:00	52	28.92 ± 11.45	23.39 kg/m²	21:31	35	30.26 ± 10.26	21.1 kg/m²	15:20
Chen, 2019 [27]	60	41.1 ± 9.65	24 kg/m²	29:31:00	159	41.97 ± 9.26	25 kg/m²	79:21:00	-	-	-	-	100	27.79 ± 7.1	-	-
Jin, 2020 [11]	100	27.63 ± 7.6	-	41:59:00	-	-	-	-	-	-	-	-	75	26.79	-	39:36:00
Li, 2024 [30]	74	27.36	-	32:42:00	-	-	-	-	-	-	-	-	-	-	-	32:43:00
Chien, 2015 [18]	34	37.1 ± 10.6	-	20:14	127	38.8 ± 10.5	-	55:13:00	-	-	-	-	-	-	-	-

A Description of the Characteristics of the Included Studies

The total number of participants included in the selected studies was 1,778, comprising 824 men and 954 women. Among these, 654 were healthy controls, 514 had schizophrenia, 149 had bipolar disorder, and 461 were diagnosed with depression. All participants were adults with a normal body mass index (BMI) in Table 1. The number of smokers is identified in Table 2.

**Table 2:** A description of characteristics of the included studies Notes: No., Number of Participants; BMI, Body Mass Index; a BMI for Depression with Childhood Trauma (CT) group; b BMI for Depression without Childhood Trauma (CT) group; M:F refers to the gender distribution; BMI is measured by kg/m². A dash (-) indicates that data were unavailable or not reported.

	Healthy group			Schizophrenia group			Bipolar group			Depression group		
Study ID	No.	Smoker	Comorbidities	No.	Smoker	Comorbidities	No.	Smoker	Comorbidities	No.	Smoker	Comorbidities
Basoglu, 2010 [29]	22	17	-	22	13	-	-	-	-	-	-	-
Sun, 2016 [28]	15	-	-	13	-	-	-	-	-	-	-	-
Lu, 2021 [25]	82	-	-	61	-	-	-	-	-	-	-	-
Wang, 2023 [31]	51	8	-	-	-	-	-	-	-	97	15	-
Li, 2021 [13]	82	-	-	-	-	-	57	-	-	74	-	-
Tsuchimine, 2019 [12]	80	10	-	80	24	-	40	8	-	80	13	-
Yu, 2023 [26]	54	6	-	54	22.0±2.2	-	52	8	-	35	7	-
Chen, 2019 [27]	60	7	30	159	33	184	-	-	-	100	-	-
Jin, 2020 [11]	100	-	-	-	-	-	-	-	-	75	-	-
Li, 2024 [30]	74	-	-	-	-	-	-	-	-	-	-	-
Chien, 2015 [18]	34	-	-	127	20	-	-	-	-	-	-	-

The pooled sample across the included studies consisted of healthy participants as well as individuals diagnosed with schizophrenia, major depressive disorder (MDD), and bipolar disorder (BD). Healthy participants (n = 654) had no history of medication use. Within the schizophrenia group (n = 514), 115 patients were first-episode and drug-naive, drawn from Lu et al. (n = 61) and Yu et al. (n = 54) [25,26]. Among the medicated schizophrenia patients, treatment regimens varied. For example, in Chien et al. [18], of 127 patients, 21 were on typical antipsychotics and 99 on atypical antipsychotics. Tsuchimine et al. reported extensive polypharmacy in 80 patients, including 52 on typical antipsychotics, 14 on atypical antipsychotics, and 17 on benzodiazepines [12]. Some studies specified particular medications, with 122 patients treated with clozapine, 20 on olanzapine, and 50 on other less obesogenic atypical antipsychotics like aripiprazole or amisulpride [27-29].

For depression (n = 461), 175 patients were first-episode and treatment-naive according to Jin et al. and Li et al. [11,30]. Medication use among treated patients was inconsistently reported: Wang et al. noted that 50 out of 97 patients (51.5%) were on medication, though the types of medication were unspecified [31]. Li et al. included 74 MDD patients but only clarified that benzodiazepines had not been used in the previous six months [13]. Tsuchimine et al. reported that 77 of 80 patients (96.2%) were on antidepressants, while Yu et al. found 18 of 35 patients (51.4%) were medicated with antidepressants [12,26].

In the bipolar disorder group (n = 149), no patients were medication-naive, and polypharmacy was common. Yu et al. described 52 patients, with 32 on mood stabilizers, 30 on antipsychotics, and 24 on antidepressants [26]. Similarly, Tsuchimine et al. reported 40 BD patients receiving treatments including antidepressants (18 patients), lithium (14 patients), and benzodiazepines (15 patients) [12]. Li et al. included 57 BD patients but did not specify medication regimens [13].

Patient Reported Outcomes, Complications, and Clinical Outcomes 

Orexin A levels in patients with schizophrenia, major depressive disorder, and bipolar disorder groups are shown in Table 3.

**Table 3:** Primary outcomes information for orexin A levels in the schizophrenia, bipolar, and major depression disorder groups compared to controls Notes: Study ID is for last name of first co-authors, year of publications; F: Female; M: Male; c: at the baseline; d: 8 weeks of clozapine treatment; e: Less obesogenic AP; f: depression with childhood trauma (CT) group; h: Depression without childhood trauma (CT) group; SE: standard error; t: Combined clozapine and less obesogenic AP; b: combined male and female values. A dash (-) indicates that data were unavailable or not reported.

	Healthy group		Schizophrenia group		Bipolar Disorder group		Major Depression Disorder group	
Study ID	Value	p-value	Value	p-value	Value	p-value	Value	p-value
Basoglu, 2010 [29]	14.18 ± 33.7	0.015	18.9 ± 40.3	0.015	-	-	-	-
Sun, 2016 [28]	168.81 ± 13.87	<0.05	160.96 ± 11.70ᶜ / 181.44 ± 17.25ᵈ	0.49	-	-	-	-
Lu, 2021 [25]	45.58±9.63 (M) / 52.42±11.94 (F) / 49.25±11.39	0.007	43.3±4.09 (M) / 42.78±4.92 (F) / 42.78±4.89	0.001 (Total) / 0.094 (M)	-	-	-	-
Wang, 2023 [31]	1.330 ± 3.140	0.861	-	-	-	-	1.230 ± 1.270 ᶠ / 1.180 ± 8.50 ʰ	0.861
Li, 2021 [13]	49.25 ± 8.65	<0.001	-	-	65.50 ± 4.03	<0.001	59.04 ± 7.39	<0.001
Tsuchimine, 2019 [12]	108.8 ± 29.1	-	99.5 ± 35.7	0.007	88.2 ± 16.8	0.010	96.2 ± 27.5	0.089
Yu, 2023 [26]	758.58 ± 215.44 (29.31 SE)	<0.001	602.56 ± 156.00 (21.22 SE)	<0.001	645.65 ± 225.91 (31.33 SE)	<0.001	602.56 ± 142.57 (24.1 SE)	<0.001
Chen, 2019 [27]	600 ± 170	<0.01	Clozapine 1.54±0.60 / 1.89±0.50ᵉ / 1650.1±591.8ᵗ	<0.01	-	-	-	-
Jin, 2020 [11]	24.13 ± 30.18	<0.001	-	-	-	-	34.26 ± 36.68	<0.001
Li, 2024 [30]	12.77	<0.001	-	-	-	-	27.20	<0.0001(F) / 0.999(M)
Chien, 2015 [18]	38.8 ± 15.5	<0.001	60.7 ± 37.9	<0.001	-	-	-	-

Schizophrenia

In this review of six studies on the schizophrenia group, both Chen et al. and Chien et al. found that orexin-A levels were slightly higher in patients receiving less obesogenic antipsychotics compared to those on clozapine [18,27]. In Chien’s study, individuals with schizophrenia exhibited higher orexin-A levels than healthy controls, with increased orexin-A associated with fewer negative and disorganized symptoms [18]. Importantly, plasma orexin-A levels were not directly linked to the weight gain liability of these medications. Similarly, Basoglu et al. found that patients with schizophrenia had higher orexin A levels compared to healthy controls [29]. On the other hand, in Sun et al., orexin release was delayed by four hours following eight weeks of clozapine treatment [28]. Lu et al. and Yu et al. observed reduced orexin A levels in individuals with schizophrenia relative to control participants [25,26]. The changes in PANSS score are shown in Table 4.

**Table 4:** PANSS score Notes: a: Baseline PANSS positive symptoms score; b: Second week PANSS positive symptoms score; c: Sixth week PANSS positive symptoms score; d: Pre-treatment; e: Post-treatment; h: Baseline PANSS negative symptoms score; s: Second week PANSS negative symptoms score; k: Sixth week PANSS negative symptoms score; m: Baseline PANSS general psychopathology score; y: Second week PANSS general psychopathology score; z: Sixth week PANSS general psychopathology score. A dash (-) indicates that data were unavailable or not reported.

	PANSS positive symptoms		PANSS negative symptoms		PANSS general psychopathology	
Study ID	-	p-value	-	p-value	-	p-value
Basoglu, 2010 [29]	21.4±4.1ᵃ / 14.9±4.5ᵇ / 11.3±4.5ᶜ	<0.001	24.5±8.5ʰ / 17.3±5.7ˢ / 16.2±7.4ᵏ	0.003	49.4±6.9ᵐ / 37.9±11.0ʸ / 30.6±13.9ᶻ	<0.0001
Sun, 2016 [28]	24.69ᵈ / 9.08ᵉ	0.019	30.46ᵈ / 12.92ᵉ	0.001	53.08ᵈ / 24.31ᵉ	0.001
Tsuchimine, 2019 [12]	13.8 ± 5.3	-	15.5 ± 6.2	-	31.3 ± 8.1	-
Chien, 2015 [18]	7.97 ± 4.38	0.968	16.19 ± 6.31	0.057	6.18±3.16 / 11.11±3.66 / 7.78±2.95	-

Chen’s study involved patients who were using antipsychotic medications [27]. In contrast, the studies by Lu et al. included only participants who were not taking antipsychotic drugs [25]. Basoglu et al. and Sun et al. included participants who had been treated with olanzapine for six weeks and clozapine for eight weeks, respectively [28,29]. 

**Figure 2:**
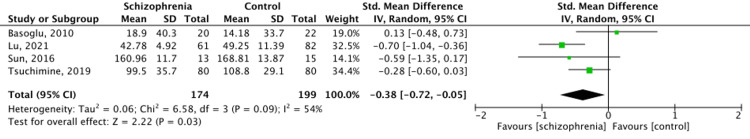
Meta-analysis schizophrenia vs. control

The meta-analysis revealed a significant decrease in orexin-A levels in the schizophrenia group compared to healthy controls (4 studies, 373 participants). The pooled standardized mean difference (SMD) was -0.38 (95% CI: -0.72 to -0.05; p = 0.03) (Figure 2). Moderate statistical heterogeneity was observed (I^2 = 54%, p = 0.09). This variability reflects the wide range of absolute values reported across different assay platforms.

**Figure 3:**
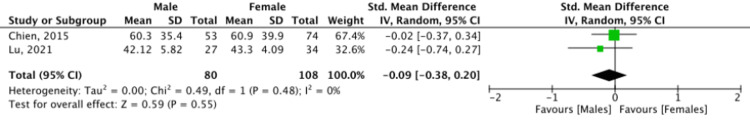
Meta-analysis of schizophrenia and sex differences

To further investigate potential sources of heterogeneity, a subgroup analysis based on gender was conducted on two studies [18,25]. However, this analysis did not reveal any significant differences in orexin-A levels between males and females (two studies, SMD = -0.09, 95% CI: -0.38 to 0.20; I^2 = 0%, p = 0.55) (Figure 3).

Depression

In this review of eight studies on depression groups, Jin et al. and Li et al., which included treatment-naive and right-handed participants, reported an increase in orexin A levels [11,30]. Wang et al. found that orexin-A levels did not differ among the three groups of depression with and without childhood trauma and healthy controls [31]. Yu et al. found that orexin levels in patients with depression were lower compared to controls, while Tsuchimine et al. reported a decrease [12,26]. Li et al. observed elevated orexin A levels in patients with schizophrenia relative to the control group [13]. Some studies, including Yu et al. and Tsuchimine et al., were excluded from the meta-analysis due to differences in clinical characteristics and inclusion criteria (e.g., use of medications, handedness) [12,26].

**Figure 4:**
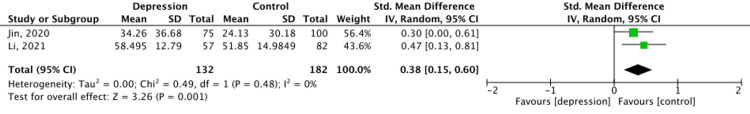
Meta-analysis depression vs. control

For the depression group, a significant increase in orexin-A levels was observed (two studies, 314 participants). The pooled standardized mean difference (SMD) was 0.38 (95% CI: 0.15 to 0.60; p = 0.001) (Figure 4). Statistical heterogeneity was absent (I^2 = 0%), indicating consistent effect sizes across the included studies despite differing absolute baseline values.

Bipolar

In this review of three studies on bipolar groups, Li et al. found an increase in orexin A levels, Tsuchimine et al. reported reduced plasma orexin-A levels in patients with bipolar disorder, and Yu et al. observed a decrease [12,13,26]. A meta-analysis was not conducted for bipolar disorder studies due to high heterogeneity among them. This variability was mainly attributed to differences in inclusion criteria. For instance, Li et al. and Yu et al. included only right-handed participants, while Tsuchimine et al. did not apply any handedness restriction [12,13,26]. Additionally, Li et al. and Yu et al. differed in other participant characteristics, such as age and educational background [13,26]. These inconsistencies made it difficult to combine the data meaningfully in a meta-analysis.

Quality Assessment and Risk of Bias

The quality assessment revealed generally robust methodological standards across the included literature, as demonstrated in Table 5.

**Table 5:** Quality assessment scores

Study (Author, Year)	Study Design	Assessment Tool	Total Score	Risk of Bias
Sun, 2016 [28]	Prospective Cohort	MINORS	20/24	Low
Basoglu, 2010 [29]	Prospective Cohort	MINORS	17/24	Low-Moderate
Lu, 2021 [25]	Case-Control	MINORS	14/24	Moderate
Li, 2021 [13]	Case-Control	MINORS	12/24	Moderate
Wang, 2023 [31]	Cross-sectional	AXIS	19/20	Low
Tsuchimine, 2019 [12]	Cross-sectional	AXIS	19/20	Low
Yu, 2023 [26]	Cross-sectional	AXIS	18/20	Low
Chen, 2019 [27]	Cross-sectional	AXIS	18/20	Low
Jin, 2020 [11]	Cross-sectional	AXIS	18/20	Low
Li, 2024 [30]	Cross-sectional	AXIS	18/20	Low
Chien, 2015 [18]	Cross-sectional	AXIS	17/20	Low-Moderate

Non-randomized studies (MINORS): among the cohort and case-control studies, scores ranged from 12 to 20 out of a possible 24 points. One prospective cohort study achieved a high score of 20, indicating a low risk of bias, while the case-control studies scored between 12 and 14, reflecting moderate methodological quality due to limitations in prospective calculation of study size and follow-up reporting.

Cross-sectional studies (AXIS): the included cross-sectional studies demonstrated high reporting quality, with AXIS scores ranging from 17 to 19 out of 20. Strengths were noted in clear aims, appropriate study design, and justified discussions, while common limitations included the lack of justified sample size calculations and non-response bias analysis.

Discussion 

To the best of current knowledge, this is the first systematic review and meta-analysis that provides insight into investigating plasma orexin-A levels across patients with schizophrenia (SCZ), bipolar disorder (BD), and major depressive disorder (MDD), synthesizing data from 11 studies involving 1,778 participants [11-13,18,25-31].

Orexin Dysregulation in Schizophrenia

The meta-analysis revealed that circulating orexin-A levels were significantly reduced in patients with schizophrenia (p = 0.03). While the effect size was small to moderate (SMD = -0.38), the direction of the effect was consistent. The moderate heterogeneity observed (I^2 = 54%) suggests that while the trend of reduced orexin is consistent, the magnitude of this reduction varies, likely due to differences in assay sensitivity or medication effects (e.g., clozapine vs. other antipsychotics).

This finding aligns with previous research highlighting the potential role of orexin deficiency in the pathophysiology of the disorder. Previously, Lu et al. highlighted the potential role of orexin deficiency in schizophrenia in support of this finding [25]. Orexin is a neuropeptide that plays a role in arousal, circadian rhythms, and wakefulness [1]. Recent evidence also implicates orexins in attention and cognitive function, with animal models of orexin deficiency exhibiting significant cognitive impairment [1,32]. Consequently, the present findings imply that the observed orexin deficiency may constitute a neurobiological mechanism underlying the cognitive deficits and negative symptoms commonly observed in schizophrenia.

Subgroup Analysis by Gender

Contrary to earlier reports suggesting gender-specific alterations, this meta-analysis observed no gender-based differences in orexin-A levels among schizophrenia patients [25]. This supports the conclusion by Li et al. that orexin-A dysregulation in this population is likely a core feature of the disorder, independent of sex [19].

Role of Antipsychotics

The systematic review indicates that patients treated with antipsychotics, particularly clozapine, exhibit lower orexin-A levels. Basoglu et al. similarly reported that patients treated with atypical antipsychotics displayed reduced peptide levels [29]. This agreement with Chen et al. suggests a potential confounding influence of medication [27]. It remains difficult to disentangle whether reduced orexin-A is an intrinsic trait of schizophrenia or a secondary effect of antipsychotic treatment, given that clozapine has been shown to disrupt circadian rhythms and delay orexin release phases.

*Orexin Dysregulation in Major Depressive Disorder* 

In contrast to schizophrenia, the meta-analysis revealed significantly elevated orexin-A levels in patients with MDD compared to controls. This result is consistent with findings by Jin et al. [11]. Given orexin’s role in emotional regulation and the sleep-wake cycle, these elevated levels may reflect a state of physiological hyperarousal often seen in depression with insomnia [1]. Clinically, this supports the mechanism of dual orexin receptor antagonists (DORAs), such as suvorexant, which improve symptoms in patients with depression by dampening this overactive signaling [33]. However, variance exists in the literature, with some studies reporting reduced levels. This discrepancy highlights the need to account for clinical heterogeneity, such as depression subtypes (e.g., atypical vs. melancholic), which may present with different arousal profiles [12,26].

Orexin Dysregulation in Bipolar Disorder

A quantitative meta-analysis was not conducted for the bipolar disorder subgroup due to extreme statistical heterogeneity (I^2 > 90%). This statistical variance likely reflects profound methodological and clinical heterogeneity, specifically differences in patient mood states (e.g., acute mania versus bipolar depression) and medication regimens (e.g., lithium versus antipsychotics) across the included studies. Consequently, these findings are summarized qualitatively. For instance, Tsuchimine et al. reported lower plasma orexin-A levels in patients with bipolar disorder, whereas Yu et al. observed significantly higher levels [12,26]. This contradiction suggests that orexin dysregulation in bipolar disorder may be state-dependent, fluctuating between manic and depressive episodes, rather than serving as a static trait marker.

Limitations

This study is subject to several limitations. First, significant clinical heterogeneity was observed, particularly regarding potential confounders such as body mass index (BMI) and smoking status, which are known to modulate orexin physiology. These variables were inconsistently reported across studies, preventing a fully adjusted meta-regression. Second, the restriction to English-language publications may have introduced selection bias. Third, the majority of included studies utilized small sample sizes (n < 100), limiting statistical power and increasing the risk of Type II errors [34]. Finally, the variability in assay methods (RIA vs. ELISA) and unit reporting (pg/mL vs. ng/L) across studies complicates the direct comparison of absolute plasma concentrations.

Future recommendations

To address these limitations, future research should employ longitudinal designs to assess orexin-A stability across different disease phases, particularly in bipolar disorder. Researchers are encouraged to standardize assay protocols and report results in uniform units (pg/mL) to facilitate valid quantitative synthesis. Furthermore, large-scale, multi-center studies that control for metabolic confounders (BMI, medication status) are necessary to confirm the diagnostic utility of plasma orexin-A biomarkers. Clinically, these results suggest divergent therapeutic avenues: exploring orexin agonists for schizophrenia-associated cognitive deficits and orexin antagonists for depression-associated hyperarousal.

## Conclusions

This meta-analysis demonstrates a divergent pattern of neuroendocrine dysregulation, characterized by significantly reduced plasma orexin-A levels in schizophrenia and elevated levels in major depressive disorder. Conversely, the high heterogeneity observed in bipolar disorder suggests that orexin-A alterations may be state-dependent (fluctuating between manic and depressive episodes) or modulated by psychotropic medications, rather than serving as a static trait marker. Clinically, these results imply distinct therapeutic avenues: potential utility for orexin agonists in addressing negative symptoms in schizophrenia, versus orexin antagonists to manage hyperarousal in depression. Future research must prioritize longitudinal designs controlling BMI and medication to validate orexin-A as a differential diagnostic biomarker.
